# Supplementary material for: Cell-death induced immune response and coagulopathy promote cachexia in Drosophila
Source: bioRxiv. 2025 Feb 10:2025.01.07.631515. Originally published 2025 Jan 8. Preprint. [Version 2] doi: 10.1101/2025.01.07.631515 (PMC11741341; doi:10.1101/2025.01.07.631515)
Supplement: Supplement 1 [file media-1.pdf]

Supplementary Table 1: List of identified proteins secreted from BirA* labeled tumorous and control guts. Column A: Flybase gene ID; Column B: Gene name; Column C: Unique Protein sequence identified in mass-spectrometry Column D: Presence of a signal peptide; Column E: Log fold change in Yki^{act} compared to BirA* control; Column F: Log fold change in Ras^{act} compared to BirA*control; Column G: Log fold change in BirA* compared to control; Column H: Log fold change in Yki^{act} compared to Ras^{act} Column I : Expression enrichment of genes in enterocytes (ECs) of Yki^{act} gut compared to control guts, derived from snRNAseq data[34]. Column J: Expression enrichment of genes in intestinal stem cells (ISCs) of Yki^{act} gut compared to control guts, derived from snRNAseq data; Column K: Protein size; Column L: Human Ortholog; Column M: Diopt Score; Column N: differential Expression in snRNAseq.

FBgn	Gene	UniProt	signalP	logFC Yki-BioID	logFC Ras1A-BioID	logFC BioID-WT	logFC Yki-Ras1A	EC (Yki/WT)	ISC (Yki/WT)	Protein size	Human ortholog(s)	DIOPT scor	snRNA Expression
FBgn0002564	Lsp1gamma	P11997	Yes	3.53	-0.65	-0.05	4.17	2.57	162.67	772 aa	–	–	yes
FBgn0033942	Cpr51A	Q7JZW0	Yes	3.3	-0.1	-0.27	3.4	11.14	130.17	144 aa	–	–	yes
FBgn0034511	GNBP-like3	A1ZBU5	Yes	2	-0.3	-0.1	2.3	6	31	152 aa	–	–	Yes
FBgn0032281	CG17107	Q9VKS2	Yes	2.83	0.66	0.14	2.17	12	4	96 aa	–	–	yes
FBgn0266420	Ote	P20240	No	2.09	0.13	-0.13	1.96	2.11	2.8	424 aa	OTOG;	1	Yes
FBgn0040321	GNBP3	Q9NHA8	Yes	2.45	0.6	-0.39	1.85	2.88	4	490 aa	–	–	yes
FBgn0035806	PGRP-SD	Q9VS97	Yes	2.17	0.31	-0.21	1.85	15.67	160	186 aa	PGLYRP1;	8	Yes
FBgn0014865	Mtk	Q24395	Yes	2.76	1.06	0.3	1.7	18	13	52 aa	–	–	Yes
FBgn0004045	Yp1	P02843	Yes	2.55	0.87	-0.11	1.68			439 aa	LIPC;PNLIP;PNLIPR	4	yes
FBgn0266405	CG45045	A0A6H2EDS4	Yes	2.42	0.75	0.23	1.68	6	2	42 aa			No
FBgn0036022	CG8329	Q9VT23	Yes	1.09	-0.58	-0.2	1.67	1	0	259 aa	CTRB1;CTRB2;CTRL	2	No
FBgn0005391	Yp2	P02844	Yes	2.5	0.85	0.03	1.65			442 aa	LIPC;PNLIP;PNLIPR	4	–
FBgn0039685	Obp99b	Q9VAI6	Yes	1.02	-0.62	-0.58	1.64	0	0	149 aa			No
FBgn0024293	Spn43Ab	A1Z6V5	Yes	1.76	0.13	-0.09	1.63	4.05	7.67	393 aa	SERPINI2;SERPINI1	5	yes
FBgn0032213	CG5390	Q9VL01	Yes	1.38	-0.25	-0.63	1.63	1.81	2	206 aa	TPSG1;TPSD1;TPSI	3	yes
FBgn0261989	CG42807	Q7JWE2	Yes	1.14	-0.48	-0.34	1.62	20	9	278 aa			Yes
FBgn0038105	yellow-f2	Q9VG08	Yes	1.05	-0.58	-0.3	1.62	5	1	452 aa	RGN;	1	No
FBgn0040735	BomT2	A1ZB62	Yes	1.45	-0.14	-0.32	1.59	5.44	9.83	76 aa	–	–	Yes
FBgn0037906	PGRP-LB	Q8INK6	Yes	1.25	-0.34	-0.11	1.59	2.97	24.73	215 aa	PGLYRP2;	10	yes
FBgn0030251	CG2145	Q9VZ49	Yes	1.84	0.26	-0.32	1.58	8	9	592 aa	ENDOU;	15	Yes
FBgn0028526	CG15293	Q9V3Y7	Yes	1.79	0.33	-0.44	1.46	6.67	37	333 aa	–	–	yes
FBgn0032836	CG10680	Q9VIQ5	Yes	2.13	0.75	-0.5	1.39	8.08	10	251 aa	–	–	Yes
FBgn0038252	BigH1	Q8MSC4	No	1.18	-0.2	-0.18	1.38	0.68	0.9	353 aa	H1-8;H1-0;	2	no
FBgn0034407	DptB	A1ZBF6	Yes	2.97	1.6	-0.08	1.37	7	3	120 aa			No
FBgn0033367	PPO2	Q9V521	No	1.85	0.47	-0.73	1.37	3	0	684 aa			No
FBgn0034468	Obp56a	Q9V8Y2	Yes	1.51	0.17	0.39	1.33	1.7	7	139 aa			Yes
FBgn0037891	CG5214	Q9VGQ1	No	0.86	-0.46	0.17	1.32	1.71	5.13	468 aa	DLST;	16	Yes
FBgn0032472	CG9928	Q9VK39	Yes	2.51	1.23	-0.17	1.29	3.79	6.14	106 aa	–	–	yes
FBgn0038535	alt	Q960Y8	No	1.05	-0.21	-0.03	1.26	2.7	5.42	874 aa	RRBP1;	4	Yes
FBgn0022959	yps	Q95RE4	No	1.32	0.08	-0.44	1.24	1.85	3.44	340 aa	YBX2;	12	yes
FBgn0031296	gNacalpha	Q9VPV7	No	1.33	0.1	-0.46	1.23	0	0	347 aa	NACA;NACA2;	8	no
FBgn0054051	CG34051	Q6IH48	Yes	1.31	0.08	-0.56	1.23	0.6	2	222 aa			No
FBgn0034512	Bbd	A1ZBU8	Yes	1.88	0.66	-0.13	1.22	8.76	9.92	245 aa	–	–	Yes
FBgn0053470	BaraA1	C0HLZ9	Yes	2.15	0.94	-0.36	1.21			257 aa			–
FBgn0260746	Ect3	Q9VGE7	Yes	1.05	-0.15	-0.51	1.2	2.27	8	637 aa	GLB1L;	17	Yes
FBgn0039308	CG11889	Q9VBU0	No	0.91	-0.27	-1.21	1.17			420 aa			–
FBgn0034470	Obp56d	Q8SY61	Yes	1.18	0.03	-0.16	1.15	1.7	1.6	131 aa			Yes
FBgn0010228	HmgZ	Q06943	No	1.15	0	-0.37	1.15	3.39	6.96	111 aa	HMGB1;HMGB2;HM	4	yes
FBgn0029715	CG11444	Q9W4J4	No	0.83	-0.32	-0.26	1.15	2.22	4	215 aa	PDAP1;	15	yes
FBgn0040582	BomBc3	Q9VD48	Yes	2.65	1.53	-0.63	1.12	12.86	31.5	98 aa	–	–	yes
FBgn0028543	NimB2	Q7KTA1	Yes	2.32	1.21	-0.34	1.12	3.38	2.8	403 aa	MEGF11;MEGF10;	3	yes
FBgn0039678	Obp99a	Q9VAJ4	Yes	0.82	-0.3	-0.08	1.12	0.63	0.83	142 aa			No
FBgn0039682	Obp99c	Q9VAI9	Yes	1.82	0.71	-0.4	1.11	2.82	3.43	151 aa	–	–	yes
FBgn0013763	Idgf6	Q23997	Yes	0.63	-0.47	0.02	1.11	1.75	3.76	452 aa	CHI3L2;OVGP1;CHI	5	Yes
FBgn0030160	CG9691	Q9W306	Yes	2.05	0.95	-0.32	1.1	4.31	12	121 aa	–	–	Yes
FBgn0004240	DptA	P24492	Yes	2.78	1.7	-0.12	1.09	6	2	106 aa			Yes
FBgn0051313	CG31313	Q8SZN1	Yes	2.06	0.98	-0.6	1.08	7.14	3.75	124 aa	CST11;CST7;CST3;	2	Yes
FBgn0262057	Spn77Ba	Q0E8C8	Yes	0.79	-0.29	-0.53	1.08	4.25	4.5	450 aa	SERPINB10;	6	yes
FBgn0012042	AttA	P45884	Yes	2.54	1.47	-0.05	1.07	15	18	221 aa	–	–	yes
FBgn0011694	EbpII	Q23982	Yes	0.93	-0.12	-0.22	1.05	3	3	66 aa			Yes
FBgn0011695	EbpIII	Q9W1C9	Yes	1.79	0.77	-0.54	1.03	2.71	4.03	126 aa	–	–	yes
FBgn0030262	Vago	Q9VZ35	Yes	1.52	0.49	-0.88	1.03	1.89	14	160 aa	–	–	yes
FBgn0031561	IM33	Q9VQT8	Yes	1.41	0.39	-0.16	1.02	3.91	5.86	82 aa	EPPIN-WFDC6;EPPI	6	Yes
FBgn0041581	AttB	Q9V751	Yes	2.41	1.41	0	1.01	6	9	218 aa			Yes
FBgn0051680	CG31680	Q8INU9	Yes	1.38	0.37	-1.11	1.01	1	0	147 aa			No
FBgn0000261	Cat	P17336	No	1.35	0.36	-0.53	0.99	2.42	6.5	3218 aa	CAT;	16	Yes
FBgn0010225	Gel	Q07171	Yes	1.71	0.74	-0.18	0.98	2.94	3.56	798 aa	GSN;	15	Yes
FBgn0032638	SPH93	Q9VJD7	Yes	1.43	0.45	-0.36	0.98	2	1	494 aa	PRSS21;TPSG1;TP	2	No
FBgn0039031	Gbp3	Q9VCT2	Yes	1.2	0.21	-0.22	0.98	2.71	3	159 aa			Yes
FBgn0041579	AttC	Q95NH6	Yes	3.44	2.47	-0.12	0.97	12	3	241 aa			Yes
FBgn0031313	CG5080	Q7K3E2	Yes	1.53	0.56	-0.37	0.97	2.6	5.4	554 aa	–	–	Yes
FBgn0034331	BomBc2	A1ZB61	Yes	1.25	0.31	-0.39	0.93	4.15	4.67	154 aa	–	–	yes
FBgn0021906	RFeSP	Q9VQ29	No	0.62	-0.31	0.2	0.93	2.4	3.96	230 aa	UQCRFS1;	15	Yes
FBgn0061515	endos	Q9VUB8	No	0.75	-0.16	-0.1	0.91	3.06	4.69	119 aa	ARPP19;ENSA;	14	Yes
FBgn0027090	GlnRS	Q9Y105	No	0.62	-0.29	-0.34	0.91	2.33	4.23	778 aa	QARS1;	17	Yes
FBgn0033451	Marc	A1Z803	No	0.6	-0.31	-0.23	0.91	1.6	3.33	340 aa	MTARC2;MTARC1;	15	Yes
FBgn0043841	vir-1	Q0E8R1	Yes	1.16	0.27	-0.63	0.89	3.24	4.4	423 aa	–	–	yes
FBgn0004687	Mlc-c	P54357	No	1.89	1.01	-0.57	0.88	3.59	6.68	153 aa	MYL6;	15	Yes
FBgn0024814	Clc	Q9VWA1	No	1.11	0.24	-0.27	0.87	3.21	7.57	219 aa	CLTA;	16	yes
FBgn0002645	Map205	P23226	No	1.31	0.46	-0.11	0.85	5.38	11.33	1185 aa	–	–	Yes
FBgn0043578	PGRP-SB1	Q70PY2	Yes	1.17	0.33	0.01	0.85	3	4	190 aa	PGLYRP2;	7	Yes
FBgn0041182	Tep2	Q9NFV7	Yes	1.05	0.2	-0.41	0.85	4.11	7.21	1420 aa	CD109;	14	Yes
FBgn0026441	ear	A0A0B4KG69	No	2	1.18	-0.32	0.82	2.82	8.56	931 aa	MLLT1;MLLT3;	11	Yes
FBgn0036106	CG6409	Q9VTC3	Yes	1.15	0.33	-0.33	0.82	4.3	12.11	360 aa	–	–	yes
FBgn0259975	Sfp87B	B4ZJA7	Yes	1.63	0.82	-0.29	0.81	1	0	87 aa			No
FBgn0037468	CG1943	Q9VI56	No	1.6	0.79	-0.55	0.81	5.24	5.9	118 aa	JPT2;	5	Yes
FBgn0033875	CG6357	A1Z9I0	Yes	1.07	0.27	-0.43	0.81	5.81	10.72	439 aa	CTSS;CTSK;	4	yes
FBgn0064237	Idgf5	Q8T0R7	Yes	0.98	0.17	0.3	0.81	3	8	444 aa	CHI3L2;OVGP1;CHI	4	Yes
FBgn0034200	Gbp2	Q7K533	Yes	1.69	0.9	-0.59	0.79	1.89	2.29	456 aa	QRICH2;	2	Yes
FBgn0034860	CG9812	Q9W1Q4	Yes	1.48	0.69	-0.64	0.79	0.44	3.2	245 aa	–	–	Yes
FBgn0036928	Tom20	Q95RF6	No	0.64	-0.15	-0.27	0.79	1.95	4.91	171 aa	TOMM20;	17	Yes
FBgn0050197	CG30197	Q8MKJ5	Yes	1.27	0.49	-0.89	0.78	1.65	15.5	113 aa	SLPI;WFDC5;WFDC	1	yes
FBgn0015299	Ssb-c31a	Q9VLR5	No	0.62	-0.15	-0.19	0.77	1.21	2.36	110 aa	SUB1;	12	Yes
FBgn0036619	Cpr72Ec	Q9VV46	Yes	1.15	0.39	-0.58	0.76	1	0	429aa	RRS1;SNRNP70;RB	1	No
FBgn0038465	Irc	Q9VEP8	Yes	0.71	-0.05	-0.41	0.76	3.32	4	697 aa	EPX;MPO;PXDN;LP	4	Yes
FBgn0024183	vig	Q9V426	No	1.52	0.77	-0.42	0.75	1.95	5.07	490 aa	SERBP1;	13	Yes
FBgn0037027	HIPP1	Q9VPB0	No	0.84	0.09	-0.24	0.75	2.31	5.23	926 aa	AUH;ECHDC2;CDY1	3	Yes
FBgn0033820	CG4716	Q7JRN6	Yes	1.66	0.93	-0.61	0.73	3.33	4.49	223 aa	–	–	Yes
FBgn0030321	CG1703	Q9VYW4	No	1.03	0.31	-0.22	0.72	1.92	7.88	901 aa	ABCF1;	15	Yes
FBgn0040968	CG14933	Q9VKE7	Yes	0.83	0.12	-0.71	0.72			77 aa	SPINK7;	4	–
FBgn0003462	Sod1	P61851	No	1.12	0.41	-0.54	0.71	2.15	3.98	153 aa	SOD1;	14	yes
FBgn0031560	CG16713	Q9VQT7	Yes	0.93	0.24	-0.13	0.69	3	5.5	82 aa	EPPIN-WFDC6;EPPI	6	Yes
FBgn0037810	sle	Q8INM3	No	0.98	0.3	-0.21	0.68	1.02	7.85	1430 aa	–	–	Yes
FBgn0034692	CG13502	Q9W295	No	1.37	0.71	-1.23	0.66	1	0	410 aa	ODAD4;	9	No
FBgn0033179	p47	Q7K3Z3	No	0.66	0	-0.14	0.66	2.71	7.33	407 aa	NSFL1C;	16	Yes
FBgn0051777	CG31777	Q8IQ06	Yes	1.43	0.82	-0.98	0.61	5.29	14.5	109 aa	PAPLN;TFPI2;COL6	1	Yes
FBgn0264922	Sumo	O97102	No	3.11	2.53	0.08	0.58	2.19	4.27	90 aa	SUMO3;	15	yes
FBgn0052521	Mnr	Q9VRF7	Yes	1.24	0.66	-0.8	0.58	4.43	4.6	388 aa	–	–	yes
FBgn0010774	Ref1	Q9V3E7	No	0.74	0.16	-0.53	0.58	3.27	8.7	266 aa	ALYREF;	15	Yes
FBgn0002930	nec	Q7JWX3	Yes	0.66	0.1	-0.58	0.57	3.45	5.33	476 aa	SERPINI1;SERPINB	8	Yes
FBgn0036058	CG6707	Q9VT69	No	0.6	0.03	-0.27	0.57	3.6	4.29	266 aa	PIP4P1;	16	Yes
FBgn0052185	edin	Q8IQR7	Yes	2.16	1.6	0.85	0.56	8.5	23	115 aa	–	–	Yes
FBgn0028936	NimB5	Q9VJU6	Yes	1.41	0.85	-0.55	0.56	2.5	0	315 aa	STAB1;STAB2;PEAI	2	No
FBgn0036035	CG18178	Q9VT38	No	0.71	0.16	-0.37	0.54	2.38	8.67	374 aa	–	–	Yes
FBgn0033351	AIMP1	A1Z7K8	No	0.69	0.17	-0.41	0.52	1.69	4.5	323 aa	AIMP1;	15	Yes
FBgn0264750	CG44008	M9PBA3	Yes	1.52	1.05	-0.64	0.47	3.46	6.44	79 aa	SPINK9;SPINK7;SP	5	Yes
FBgn0263198	Acn	Q9VJ12	No	0.94	0.47	-0.56	0.47	2.43	4.97	739 aa	ACIN1;	10	Yes
FBgn0003721	Tm1	P49455	No	0.88	0.41	-0.21	0.47			282 aa	TPM1;	15	–
FBgn0052190	NUCB1	Q9VVK7	Yes	1.71	1.25	-0.14	0.46	3.05	10.94	569 aa	NUCB2;	14	Yes
FBgn0033631	Sod3	Q7JR71	Yes	1.57	1.11	-0.42	0.46	1.06	3.07	243 aa	SOD1;	8	Yes
FBgn0000547	ed	Q9VQW7	Yes	0.66	0.22	0.45	0.44			1332 aa	NPHS1;	3	–
FBgn0262103	Sik3	A1ZBC4	No	0.81	0.4	-0.57	0.41	2.66	6.13	1471 aa	SIK3;MARK4;	4	Yes
FBgn0287873	zip	Q99323	No	1.75	1.37	-0.11	0.39			2024 aa	MYH10;	15	–
FBgn0038914	fit	Q9VD66	Yes	1.15	0.76	-0.05	0.39	0.89	0.64	121 aa			No
FBgn0027599	CG2790	Q9W0X8	No	0.67	0.29	-0.21	0.39	1.89	6.15	540 aa	DNAJC21;	16	Yes
FBgn0000719	fog	P40795	Yes	2.44	2.06	-0.28	0.38	8.55	6.21	730 aa	NECAP2;	1	Yes
FBgn0026721	fat-spondin	Q7KN04	Yes	0.73	0.35	-0.27	0.38	2	3.5	763 aa	SPON1;	12	Yes
FBgn0050069	Sdb	A1Z9M5	No	0.81	0.44	-0.18	0.37	2.43	4.38	4012 aa	SAXO2;SAXO1;MU	1	Yes
FBgn0261260	mgl	A8JTM7	No	0.73	0.36	-0.14	0.37			4769 aa	LRP2;	14	–
FBgn0086408	stl	Q9W1Z6	Yes	1.07	0.73	-0.22	0.34	3.04	24.33	1136 aa	ADAMTS6;ADAMTS	4	Yes
FBgn0039219	CG13630	Q9VC48	No	0.65	0.34	-0.42	0.31	1.41	7.5	374 aa	METAP1;	16	Yes
FBgn0033265	coil	A1Z7A8	No	0.93	0.64	-0.26	0.29	2.52	5.18	633 aa	–	–	Yes
FBgn0010772	Xe7	Q7KTT0	No	0.75	0.49	-0.31	0.26	3.4	4.77	783 aa	AKAP17A;	13	Yes
FBgn0035542	TP53INP	M9NFM7	No	0.91	0.66	-1.15	0.25			417 aa	TP53INP1;	7	–
FBgn0030054	Caf1-180	Q9W3D1	No	0.76	0.56	-0.47	0.2	2	5	1183 aa	CHAF1A;	11	Yes
FBgn0034753	CG2852	Q9W227	Yes	1.53	1.35	-0.22	0.18	1.92	5.62	205 aa	PPIB;	15	Yes
FBgn0010434	cora	Q9V8R9	No	0.69	0.52	-0.39	0.17	4.14	8.3	1696 aa	EPB41L3;EPB41L1	13	Yes
FBgn0039869	Tbca	Q9V9U7	No	1.45	1.28	-0.65	0.16	2.73	5.26	110 aa	TBCA;	17	Yes
FBgn0045862	btz	A0A0B4KH64	No	1.23	1.08	-0.36	0.14	2.94	12.25	806 aa	CASC3;	11	Yes
FBgn0033814	Qsox1	Q7JQR3	Yes	0.64	0.5	-0.18	0.14	6.29	4.62	637 aa	QSOX1;QSOX2;	16	Yes
FBgn0031381	Npc2a	Q9VQ62	Yes	1.92	1.79	-0.09	0.13	11.79	11.31	148 aa	NPC2;	16	Yes
FBgn0033788	CG13323	Q4V4H7	Yes	1.49	1.36	0.04	0.13	4.89	10.91	112 aa	–	–	Yes
FBgn0032243	Klp31E	Q9VKW4	No	0.67	0.54	-0.48	0.13	2.77	4.31	1048 aa	KIF21A;	15	Yes
FBgn0261641	CG42724	A0A0B4KFB8	No	0.7	0.6	-0.14	0.1	2.57	4.46	1123 aa	TCERG1;	13	Yes
FBgn0032283	CG7296	Q8SXV4	Yes	0.92	0.84	0.03	0.08	4.33	2	161 aa	HNRNPA2B1;COL21	1	Yes
FBgn0031217	Creld	Q9VPJ0	Yes	0.64	0.56	0.35	0.08	1.47	5.24	374 aa	CRELD2;	14	Yes
FBgn0067903	IM18	P82701	Yes	1.19	1.12	0.97	0.07			71 aa			–
FBgn0036847	CNPYb	Q9VVW7	Yes	1.71	1.65	0.4	0.06	1.82	22	221 aa	CNPY4;	15	Yes
FBgn0032773	fon	Q9VIX7	Yes	1.3	1.25	-0.59	0.05	7.67	15.67	574 aa	NUP98;NCOA5;	1	Yes
FBgn0085446	CG34417	M9PDW8	No	0.65	0.61	-0.42	0.05			4925 aa	SMTN;	7	–
FBgn0036745	CG7484	Q9VVJ7	Yes	1.15	1.12	-0.36	0.03	2.46	9.33	178 aa	SELENOF;	15	Yes
FBgn0025740	PlexB	Q9V4A7	No	1.12	1.11	-0.25	0.02	3.21	5.01	2051 aa	PLXNA1;PLXNA2;	9	Yes
FBgn0004656	fs(1)h	P13709	No	0.64	0.64	-0.17	0			2046 aa	BRD4;BRD3;	11	–
FBgn0033663	ERp60	Q3YMU0	Yes	1.16	1.17	-0.1	-0.01	2.28	8	489 aa	PDIA3;	17	Yes
FBgn0039261	Ythdf	Q9VBZ5	No	0.53	0.61	-0.36	-0.08	2.23	7.74	700 aa	YTHDF1;	13	Yes
FBgn0030734	ERp44	Q95TL8	Yes	1.14	1.23	-0.22	-0.09	1.78	6.56	412 aa	ERP44;	17	Yes
FBgn0035917	Zasp66	Q8IQB4	No	0.79	0.88	-0.52	-0.09	3.23	5.59	326 aa	PDLIM7;LDB3;PDLI	3	Yes
FBgn0027588	GCS2alpha	Q7KMM4	Yes	0.9	1	-0.13	-0.1	2.03	7.46	924 aa	GANAB;	16	Yes
FBgn0040308	Prx4	Q9V3Q4	Yes	0.63	0.73	-0.15	-0.1	1.54	22.75	242 aa	PRDX4;	16	Yes
FBgn0259984	kuz	Q9VJW9	Yes	1.13	1.24	-0.28	-0.11			1238 aa	ADAM10;	14	–
FBgn0034923	Upf3	Q9W1H3	No	0.51	0.63	-0.73	-0.11	2.04	18	492 aa	UPF3A;UPF3B;	14	Yes
FBgn0004598	Fur2	P30432	No	0.64	0.76	-0.17	-0.12	3.14	4.53	1679 aa	PCSK5;	12	Yes
FBgn0030787	CG9609	Q9VXB4	No	0.71	0.85	-0.63	-0.14	1.27	2.8	403 aa	GTF3A;	10	Yes
FBgn0039229	Saf-B	Q8IMV6	No	0.53	0.68	-0.82	-0.15	2.94	4.19	928 aa	SAFB;SLTM;	13	Yes
FBgn0259785	pzg	Q9VP57	No	0.54	0.73	-0.23	-0.18	2.85	5.95	996 aa	ZNF462;	2	Yes
FBgn0031089	aspr	Q9W5X1	Yes	0.46	0.65	-0.57	-0.18	3.33	8.67	440 aa	DNER;EDIL3;	1	Yes
FBgn0002868	MtnA	P04357	No	1.45	1.64	-1.27	-0.19	3.68	6.34	43 aa	MT1B;MT1H;MT2A;	1	Yes
FBgn0004647	N	P07207	No	0.82	1.04	0	-0.22	3.52	4.77	2693 aa	NOTCH1;NOTCH2;N	12	Yes
FBgn0037718	P58IPK	Q9VHA8	Yes	0.44	0.68	0.38	-0.24	0.98	6.43	498 aa	DNAJC3;	15	Yes
FBgn0002781	mod(mdg4)	Q86B87	No	0.4	0.65	-0.43	-0.25	2.48	4.14	540 aa	BTBD18;	3	Yes
FBgn0046214	vig2	Q9VBX3	No	0.69	0.96	-0.38	-0.27	1.56	4.43	443 aa	SERBP1;	13	Yes
FBgn0038043	CG17202	Q9VG76	No	0.61	0.89	-0.7	-0.28	2.05	2.74	181 aa	MYCBP;	7	Yes
FBgn0038842	hdly	A8JR58	Yes	2.31	2.6	-0.42	-0.3	34.64	28.85	377 aa	–	–	Yes
FBgn0058160	CG40160	Q95RS6	Yes	0.58	0.89	-0.38	-0.3	3.45	11.05	421 aa	TPSAB1;	4	Yes
FBgn0031030	Tao	Q0KHQ5	No	0.34	0.64	-0.61	-0.3	2.87	4.31	1039 aa	TAOK1;	15	Yes
FBgn0053861	His1:CG33861	Q4AB54	No	0.35	0.67	-0.07	-0.32			256 aa	H1-4;H1-5;H1-2;	8	–
FBgn0053858	His1:CG33858	Q4AB54	No	0.35	0.67	-0.07	-0.32				H1-4;H1-5;H1-2;	7	–
FBgn0053855	His1:CG33855	Q4AB54	No	0.35	0.67	-0.07	-0.32				H1-4;H1-5;H1-2;	8	–
FBgn0260456	CG4806	Q9W124	No	0.93	1.28	-0.87	-0.35	1.43	4.94	657 aa	RBM28;	14	Yes
FBgn0043903	dome	Q9VWE0	Yes	0.34	0.69	-0.07	-0.35	3.06	9.12	1282 aa	PTPRQ;	2	Yes
FBgn0037930	CG14715	Q9VGK3	Yes	1.05	1.41	0.17	-0.36	2.02	7.46	138 aa	FKBP2;	14	Yes
FBgn0286818	Pdi	P54399	Yes	1.08	1.46	0.01	-0.37	2.42	7.55	496 aa	P4HB;	17	Yes
FBgn0031016	kek5	Q9VWI6	Yes	0.53	0.91	-0.45	-0.37			931 aa	SLITRK4;LRRC24;	3	–
FBgn0033526	Caf1-105	A1Z898	No	0.32	0.69	-0.35	-0.37	1.88	6.67	747 aa	CHAF1B;	15	Yes
FBgn0051908	CG31908	Q8T9H8	No	0.82	1.21	-0.89	-0.39	2.23	5	392 aa	SZRD1;	8	yes
FBgn0033741	CG8545	A1Z909	No	0.73	1.13	-0.36	-0.39	1.84	5.13	891 aa	NOP2;	13	Yes
FBgn0026199	myo	Q9V4F4	No	1.1	1.51	-0.03	-0.41	2.56	4.11	598 aa	MSTN;	10	yes
FBgn0003567	su(Hw)	P08970	No	0.22	0.63	-0.07	-0.41	2.66	5.79	941 aa	ZNF358;ZNF746;ZB	2	Yes
FBgn0261618	larp	Q9VAW5	No	0.23	0.65	-0.47	-0.42	3.06	4.17	1673 aa	LARP1B;	14	Yes

Supplementary Table 2: List of primers used for quantitative PCR.

	**Forward**	**Reverse**
fondue	ACTCTGCTCTGGGAAAACTCG	TCTCAACGGCACCACCTAATC
tiggrin	TCTGTCAGGGCTACGAGACC	GAGTTGTGGCACTGTTTGTCC
Eig71eE	CTAACTGTGGTCTGCTTAGTGG	CAACGCTTTCTCAATTACCTCCA
Idgf3	AGCCCTACAATTCTGCACCC	CTGCTCAAGGTCCAATCGCTT
Eig71Ee	CTAACTGTGGTCTGCTTAGTGG	CAACGCTTTCTCAATTACCTCCA
fbp1	ATCGTGGCGGCATTGATAAGG	CGAAGGGTGTCAAAGTCCTG
hemomucin	AGGTCATCAAGCTAACGTCCA	TGTTGCCCTGCGTATCAAAGG
hemolectin	TGGTTATGGCGGGATAAAGACG	GTTGCCCTGACTTCCCTGG
GNBP-like3	TCTGTTCCTAgtcgCAATTTCC	GGTGAGTTGACCTTGACGGT
PGRP-lf	CACCCAGTGGGAAGTACCC	GTTCGCATCCTTCGGTTGC
tep-3	TTCCCGCCTTAAGAACTGACA	CCGTCTGAACCAAAACCGTA
Glutactin	CGGAGACCCGATATGCACAG	TACCCAAGAAAGCGTTCACTG
ppo1	TTGGAACTGCCCGATTCCTTC	TTCAGATCCACGTCCTTAGAGAA
ppo2	GAGGAGTCTTTTGTGGTGCAG	GGTGAAGGTTGATGCCCAGA
ppo3	ATCTTCACCAAAATGCAGACCG	TCGAgtcgATAAAGCGATCCG
sp7	GTTGTAGGAATCCCAACCAGA	CTCCATCGAGGCACTGTGAG
puc	TCCGGCGGTCTACGATATAGAAA	AGCAATAGATGCGGGAAAA
d-jun	ACCTGAACACATCCACCCC	ATCCGGTGAGTTGATGACCAG
lsp1gamma	GCCTGTGTGACTGCCTTTAG	AGAGGCTCATCAATACGGTGA
AttA	CACAACTGGCGGAACTTTGG	AAACATCCTTCACTCCGGGC
DptB	ATGCATTTCACCGCTAGTCT	TGCCAGTGGTTCAGGCTG
metchnikowin	ATGCAACTTAATCTTGGAGCGA	GACGGCCTCGTATCGAAAATG

drosomycin	GATGCCGACTGTCTCTCTGG	GACAGGTCTCGTTGTCCCAG
attacin D	ATGGAATGTCAGGCTTCAGGA	CCTGGAGTGGAGGCGAATAC
diptericin A	TACCCACTCAATCTTCAGGGAG	TGGTCCACACCTTCTGGTGA
imd	TCAGCGACCCAAACTACAATTC	TTGTCTGGACGTTACTGAGAGT
AttB	GCAATGGAGCTGGTCTGGAT	CCGATTCCTGGGAAGTTGCT
Dorsal	ATGTTTCCGAACCAGAACAATGG	CCGTTGTAGTTGAGGCTCTGT
Drosomycin B	GATGCCGACTGTCTCTCTGG	GACAGGTCTCGTTGTCCCAG
Defensin	CTGCAGCATAGCCGCCAGA	GCCGCCTTTGAACCCCTTGG
Drosocin	TTTTCCTGCTGCTTGCTTGC	GGCAGCTTGAGTCAGGTGAT
AttC	CGCCACCCAGAATCTACAGG	CTTAGGTCCAATCGGGCATCG
GNBP-like	TCTGTTCCTAgtcgCAATTTCC	GGTGAGTTGACCTTGACGGT
Spaetzle	GCGATTCCTTTGCAGGAGC	AATTAACTGCCAGGTgtcgTC
Toll	ATCTGAAGCATCCTTCGgtcg	GTTAGCCTAAACGTGGGATTCTC
Pelle	TGCAGCAGAGCTACAACGAA	CAGGATATTgtcgTGCCGGA
Dif	GGAGCCGACAAGCAATATAATCC	GTAGTTGCACACTTCGATGGT
Cactus	ATGCCGAGCCCAACAAAAG	CGCTAGTGGCTAGTGAGGAC
relish	CTTCCCGGAGGTTACACTGTG	GTGGGCTGTCCAAGTTAGTTT
edin	CAAGTGGgtcgGGAGGCTA	TCCGATTGTAgtcgAAATTCCG
Bombc3	TGGTGAATGGCGTCTGTCTG	ACCACATTACCATCGCCAGG
vago	AAGCGATTCCTTATCGACCCT	GATCCTCTCGCGTGAAGACTT
eiger	AGCTGATCCCCCTGGTTTTG	GCCAGATCGTTAGTGCGAGA
eiger	AGCGAGTCGTCGATAATCTCC	GCATTCTCGTACTCCTTTTGG
spz	GACACCTGGCAGTTAATTGTCA	CGAAGTCACAGGGTTGATCCG
PGRP-LC	AGGCCGTCACAGTTACAGTG	GTGGTGGCCAGTACGATACC
PGRP-SD	GACAGCATGGAAACTCCCTTG	GTTTTGCAGATTTTGCATGTGC

PGRP-SA	ACGGGCATAGCCTTTATCGG	TAATCCTCGCTCAGCTCACC
IDGF5	CAGAGGTTGGAAAACTGGTGT	GTTCAGCCAGCGACATTTGG
IDGF6	ATTCCGCCAGTTTCGTCAAGG	CGTAGACCAGATAGTCGCAGAA
RP49	ATCGGTTACGGATCGAACAA	GACAATCTCCTTGCGCTTCT
GAPDH	CCAATGTCTCCGTTGTGGA	TCGGTGTAGCCCAGGATT

Supplementary Table 3: List of primers used for chromatin immunoprecipitation.

	FORWARD	REVERSE
RP49	ATCGGTTACGGATCGAACAA	GACAATCTCCTTGCGCTTCT
*Act5c*_Promoter	GTGCAGATAGCAGTAAACGTAAGC	CCCCAACTACTCATTGTATGCC
**Sd_ChIP**		
*egr*_Promoter	TTACACAAAGTAAACAGCGCAGGC	GAACTGAGAACGGGAGCG
*egr*_intron	GCAAACTCTGGCTAGGGTATCTC	GTATGTCCCACACAATAACCAC
*Pvf1*_intron	GCAACAACAACAAGACGGC	CTTTTCATCGCTCTCCCTCC
*Impl2*_intron	CGAGCATTCGCGCACTTTAGAG	GGGGAAAACGCAACAGGTTGC
*upd3*_promoter	GCGCGCGGTGAAATTTAGAC	CTCGCAGACACGGCGATTC
**Rel_ChIP**		
*Pvf1*_promoter	GATACCTGAGGCATGGATATATGC	CTTTGGCGAAATCTGATCAGC
*Impl2*_Intron	CGACGTTGGCAGAGAAAGAG	GGAGAGTCGCGAGTTCAAATG
*Impl2*_promoter	CTTTGTTAGCGCTGAGGAGCC	CAATCTGTAGGTGGCGCGT
*upd3*_promoter	CTGTCTTATCACCGCCTAGTCAAG	GCACTCGAAATGAGTGAGCATAG
**dl_ChIP**		
*Pvf1*_promoter	GCTCTCCCAGCGTTAACTGTTAAC	CATTGCGCTCTCCGCTCTGTA

Impl2	GTGCGTGTGTATGTGTGAGTG	GCTTCCCATCTGTGTGAGTATG
*upd3*_upstream	CGCTCAGCTGTGCTTTTTTATG	GAAGCGGAAGAGAGACAGGGAG
*upd3*_upstream	CGCTCAGCTGTGCTTTTTTATG	CCGCTCGAATCGCCAAAG

Supplementary Figure 1

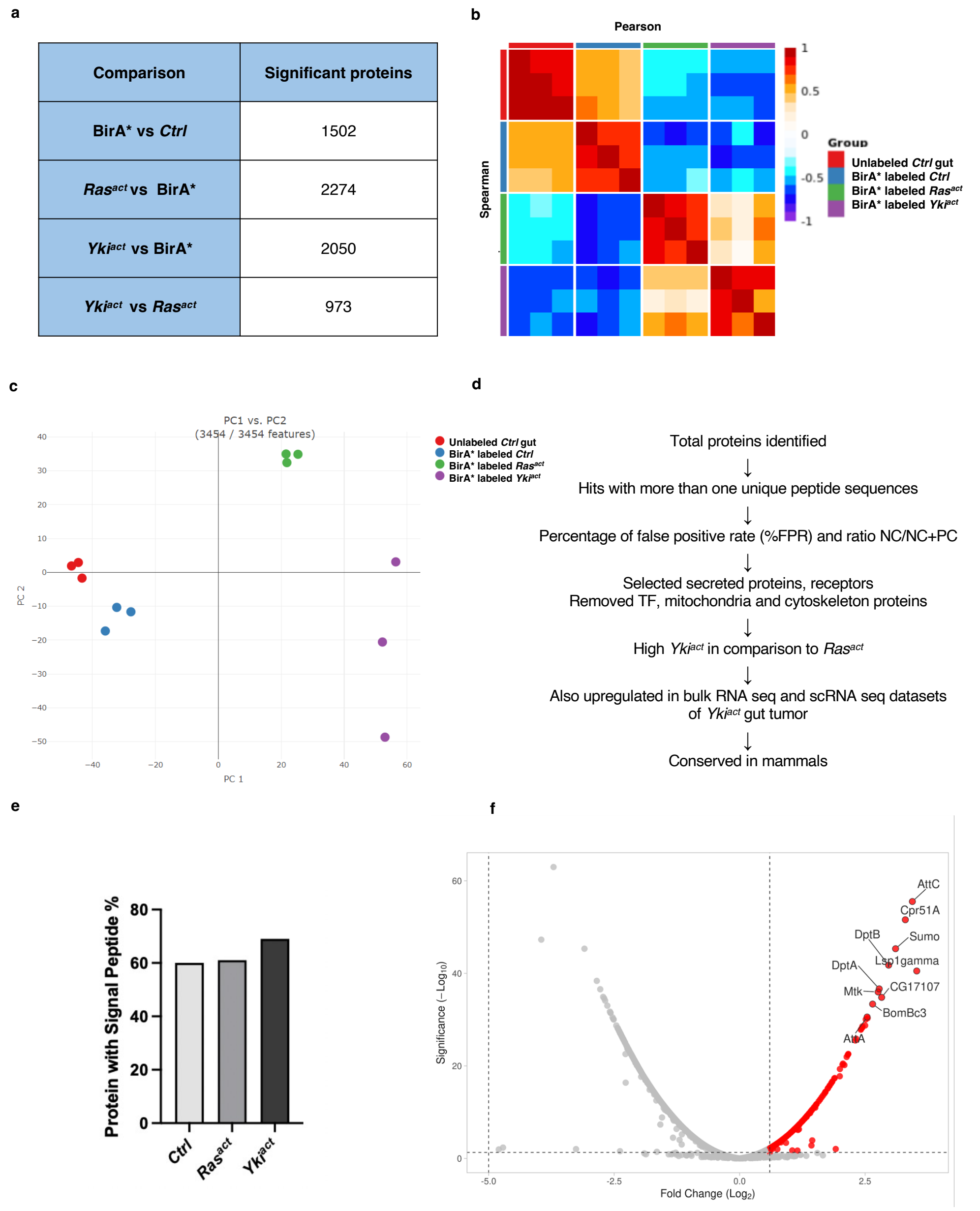

Supplementary Figure 1. Identification and analysis of Yki^{act} gut-derived proteins in hemolymph of adult flies.

(a) Number of significant proteins identified in each sample (adjusted p-value < 0.05).

(b) Triplicates of the four samples analyzed for MS showing good Spearman and Pearson correlation.

(c) Principal component analysis showing good correlation among different replicates.

(d) Filters applied to narrow down the list of proteins identified by MS.

(e) Percentage of identified hits with signal peptide sequence in each sample. Cntrl is *EGT>BirA*, Ras*^{act} is *EGT>Ras*^{act} *+BirA**, and *Yki*^{act} *is EGT>Yki*^{act}*+BirA**

(f) Volcano plot to show top 10 hits identified in Yki^{act} +BirA* gut secretomes compared to BirA* only.

Supplementary Figure 2

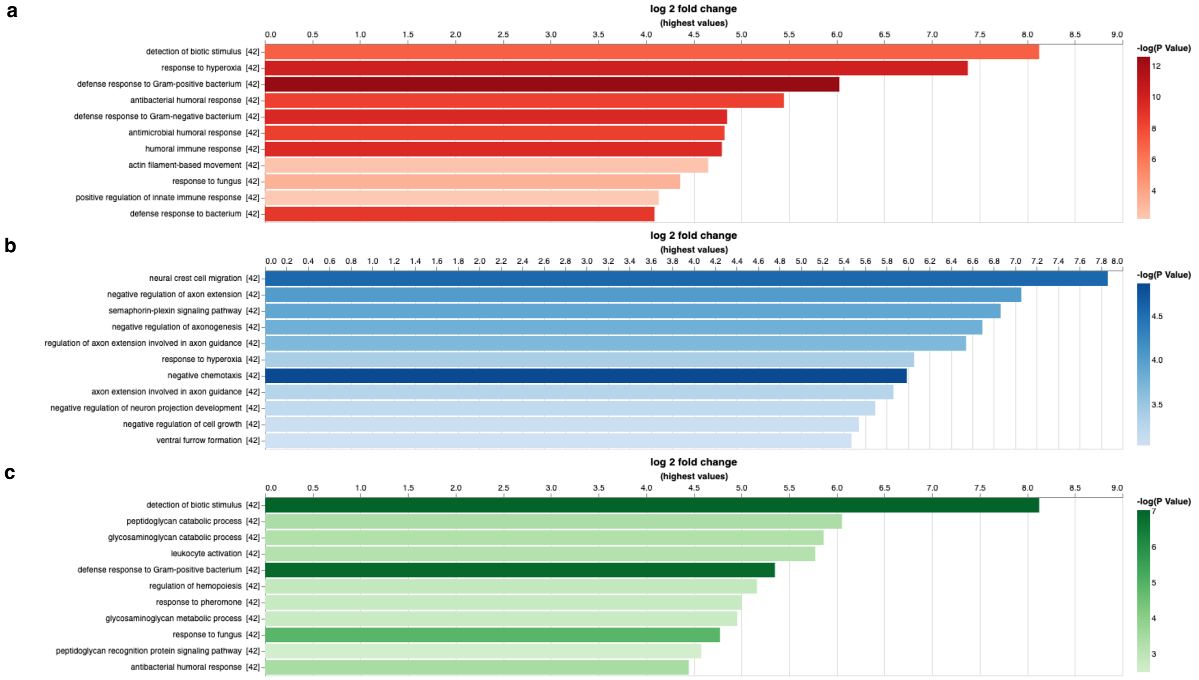

Supplementary Figure 2. Pathway enrichment analysis of top 100 proteins identified in Yki^{act} (a), Ras^{act} (b) and in both Yki^{act} and Ras^{act} (c) secretomes.

a. modERN analysis

TF	Gene	Peak Count	Location
Rel	*upd3*	2	Upstream
Rel	*upd3*	1	Intron
dl	*upd3*	1	Upstream
dl	*upd3*		Intron
Dif	*upd3*	2	Upstream
Rel	*Impl2*	3	Upstream
Rel	*Impl2*	2	Intron
Rel	*Impl1*		Upstream
dl	*Impl2*	1	Intron
Dif	*Impl2*	1	Upstream
Dif	*Impl2*	1	Intron
Rel	*Pvf1*	1	Intron
dl	*Pvf1*	1	Upstream
dl	*Pvf2*	1	Upstream
Dif	*Pvf1*	1	Upstream
Dif	*Pvf2*	1	Upstream

b. AlphaFold 3 analysis

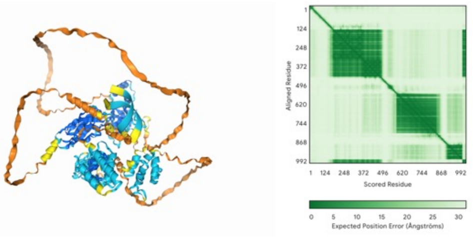

(i) Rel-*upd3* ipTM=0.92 pRM=0.41

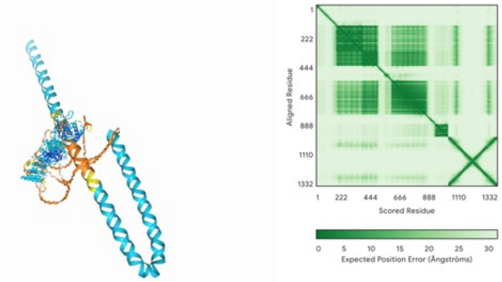

(ii) Rel-*Impl2* ipTM=0.26 pRM=0.48

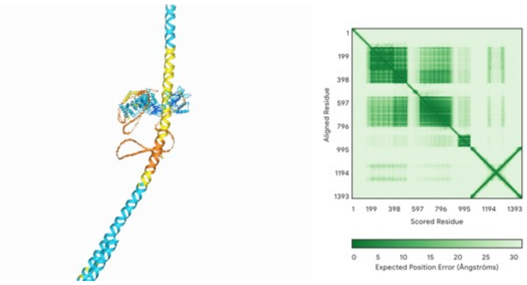

(iv) Rel-*pvf1* ipTM=0.22 pRM=0.42

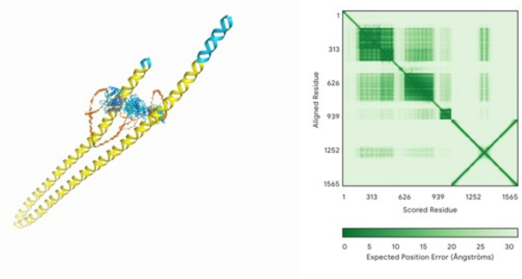

(iii) Rel-*Impl2*_prom ipTM=0.24 pRM=0.39

Supplementary Figure 3: Analysis of NFκb and Sd transcription factor binding sites

(a-b) NFκb transcription factor binding: (a) modERN analysis shows binding of NFκB transcription factors—Rel, dl, and Dif—to the regulatory sequences of *Pvf1*, *Impl2*, and *upd3*. (b) AlphaFold 3 analysis predicts binding of Rel to the regulatory regions of *upd3* (i), *Impl2* (ii, iii), and *pvf1* (iv). (c-d) Sd binding on *egr*: (c) modERN analysis suggests Sd binding to the regulatory sequences of *egr*. (d) AlphaFold 3 analysis indicating potential Sd binding to the *egr*.

Supplementary Figure 4

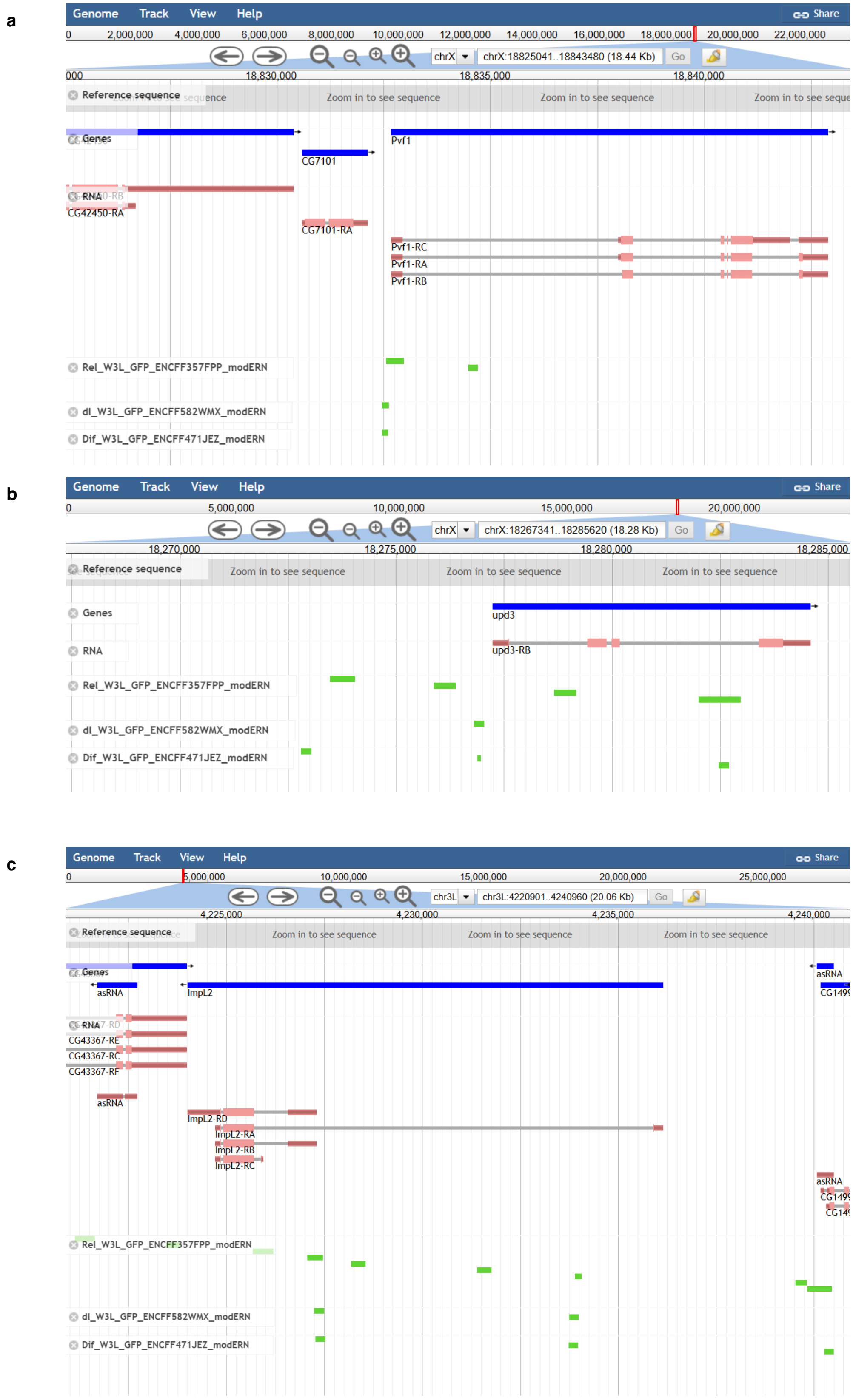

Supplementary Figure 4: Bioinformatics analysis using Chip-seq datasets from modERN. Chip-seq datasets for NFkB transcription factors from modERN near (a) *Pvf1*; (b) *upd3*; and (c) *ImpL2*.

Supplementary Figure 5

a

TF	Gene	Peak Count	Location
Sd	*Eiger*	4	Intron
Sd	*Eiger*	1	Upstream

b

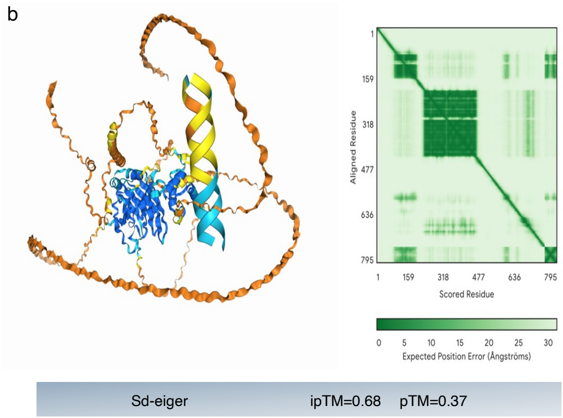

c

Gene Expression Counts

Gene: egr

Sample Name	00_indirect_flight_muscle	01_follicle_cell	07_enterocyte	08_intestinal_stem_cell
8-day WT	13	99	56	60
8-day Yki	65	89	236	223

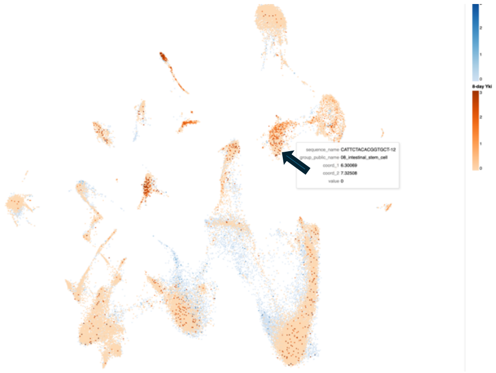

d

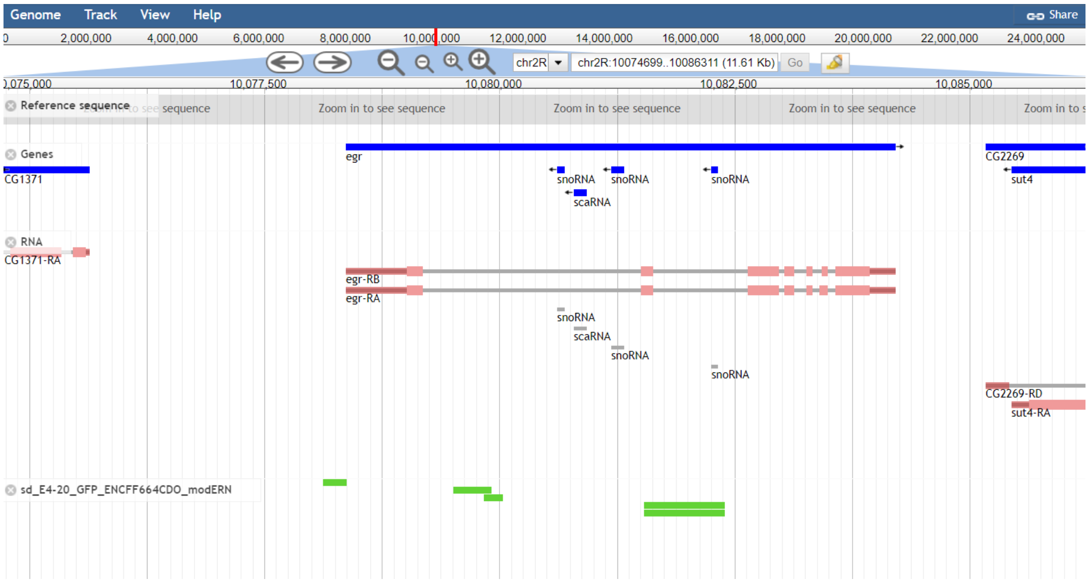

Supplementary Figure 5: Analysis of Sd transcription factor binding sites on *egr* regulatory sequence. (a) modERN analysis suggests Sd binding to the regulatory sequences of *egr*. (b) AlphaFold 3 analysis indicating potential Sd binding to the regulatory regions of *egr*. (c) snRNAseq analysis from Yki^{act}-whole body snRNAseq data[34] shows enrichment of *egr* gene expression in ISCs and EBs. (d) Bioinformatics analysis using Chip-seq datasets from modERN for Sd indicate peak signals near *egr*.
